# Oncolytic Virus Immunotherapy: Showcasing Impressive Progress in Special Issue II

**DOI:** 10.3390/biomedicines9060663

**Published:** 2021-06-10

**Authors:** Zong-Sheng Guo

**Affiliations:** UPMC Hillman Cancer Center and Departments of Surgery, Microbiology and Molecular Genetics, University of Pittsburgh School of Medicine, Pittsburgh, PA 15213, USA; guozs@upmc.edu

Cancer immunotherapy has recently become the most promising strategy for hard-to-treat, advanced-stage malignancies [1,2]. Immune checkpoint blockade (ICB), cancer vaccines, and adoptive T-cell therapy are a few hot topics. Equally hot is the development of oncolytic viruses (OVs) for cancer immunotherapy.

Many clinical studies have been conducted on OVs, and 119 papers have been reported based on 97 clinical studies on 3233 cancer patients over the last 20 years [3]. Since the approval of T-VEC for the treatment of advanced melanoma in 2015, the most exciting development is probably the combination of T-VEC with ICB for advanced melanoma, for which the overall response rate was up to 67% [4,5]. Most human cancers are quite cold and do not respond to ICB-type immunotherapy with good efficacy [6]. However, OVs can transform cold tumors to hot [7,8] (Figure 1). Thus, it may be one of most suitable agents for combination immunotherapy for cold cancers. Rational combination regimens involving OVs have a bright future in cancer immunotherapy [9,10].

Since the launch of the original Special Issue (I) on “Oncolytic Viruses as a Novel Form of Immunotherapy for Cancer,” with 15 articles in this journal in the years 2016 and 2017 [11], similar Special Issues on OVs have been launched by other journals, including *Cancers*, *Pathogens*, *Frontiers in Oncology/Immunology*, and *Human Gene Therapy*. This indicates exciting developments in this fast-advancing field. Considering these advances, last year we launched this Special Issue II with Dr. Bartlett and I as Guest Editors. Altogether, 11 scientific contributions cover some cutting-edge research areas in the field.

## 1. Original Research Articles: Significant Progress on Various Fronts

The five original research articles cover three well-studied types of OVs, including adenovirus, herpes simplex virus (HSV), vaccinia virus (VV), and one rarely studied OV, rotavirus. Summarily, the authors have explored new OVs by arming them with an apoptosis inducer, immune-stimulatory cytokines for enhanced antitumor immunity, a mutant thymidine kinase gene for improved safety, and finally, a novel methodology that enables visualization of the dynamics of viral genomes of VV and quantification in living cells.

Wang and his team studied the therapeutic effects of an armed oncolytic adenovirus called CD55-Smad4 on the inhibition of colorectal cancer both in vitro and in tumor models in vivo [12]. Smad4 is a key protein in the transforming growth factor-β signaling pathway. In many cancers, mutation or low expression of Smad4 switches its function from tumor inhibition to tumor promotion [13]. The take-home lesson from this study is the title of the article, that CD55-Smad4 can suppress cell proliferation, metastasis, and tumor stemness in colorectal cancer by regulating the Wnt/beta-Catenin signaling pathway.

Chouljenko, Jia, and their colleagues constructed a new oncolytic HSV (VG161) that encodes recombinant genes for IL-12, IL-15 (superagonist), and a fusion protein capable of disrupting PD-1/PD-L1 interactions [14]. Initial in vitro testing showed that the virus replicates efficiently and exhibits robust cytotoxicity in multiple tumor cell lines. Moreover, the encoded Th1 cytokines and the PD-L1 blocking peptide work cooperatively to boost immune cell function. Therapeutic studies demonstrated superior efficacy when compared to a parental backbone virus. In summary, VG161 can stimulate a robust anti-tumor immune response and improved efficacy without sacrificing safety.

Hwang and his team built on their previous work and replaced the thymidine kinase (tk) gene of Western Reserve (WR) strain VV by A167Y-mutated HSV-tk (HSV-tk418m) to alter nucleoside selectivity from broad spectrum to purine exclusive for potential high safety that could be regulated by a pharmaceutical [15]. The resulting OV, named WOTS-418, was significantly attenuated in normal cells, but its replication and cytotoxicity were similar to that of wild-type WR VV in cancer cells. Overall, WOTS-418 displayed robust oncolytic efficacy and pharmacological safety, and it may be useful for human clinical study in the future.

Erbs and team invented a new approach that enables visualization and quantification of viral genome dynamics of VV in living cells [16]. In the study, they incorporated the ANCH target sequence and the OR3-Santaka gene in the double-deleted VV. Quantitative analysis of viral infection kinetics and viral DNA replication allow rapid and efficient identification of inhibitors and activators of oncolytic activity. This is a very useful methodology to facilitate the future development of novel oncolytic viruses, as well as the safety and potency of these novel anti-tumor agents.

Viruses of the rotavirus genus have not been explored as OVs until recently. Acosta and colleagues investigated the potential of Rotavirus Isolate Wt1-5 for its oncolytic activity in vitro in human acute lymphoblastic leukemia cells (cell line Reh) [17]. The authors made two key observations. First, Rotavirus Isolate Wt1-5 can utilize cell-surface proteins such as heat shock proteins 90, 70, 60, and 40, Hsc70, PDI, and integrin β3 as receptors for attachment and infection. Secondly, the virus can induce apoptosis in these leukemia cells upon viral infection. Therefore, it is a candidate OV worth further exploration.

## 2. Review Articles: Exciting Progress in Various Areas in the Field

Six review articles provide excellent overviews and in-depth discussions on specific topics regarding current progress and future challenges.

Schirrmacher contributed two review articles on some fundamental mechanisms of cancer vaccine and oncolytic immunotherapy [18,19]. The first review focused on the topic of how to achieve long-term protective anti-tumor immunity mediated by virus-infected cancer vaccination and the underlying mechanisms [18]. One key message is that cancer vaccines should instruct the immune system on relevant cancer targets and provide signals for innate immunity activation. In this case, viral infection would strengthen these signals and thus elicit long-term immune memory and protection. The second article argues that cancer vaccines and OVs can exert profoundly lower side effects than other systemic therapies in cancer patients, and Schirrmacher provides a comparative analysis to support this statement [19].

Guo and collaborators reviewed another promising strategy by arming an OV with bi-specific or tri-specific antibodies that promote interaction between cancer/cancer stromal cells with either T or natural killer cells, enabling immune cell activation and tumoricidal activity [20]. One big advantage of this approach is bypassing the need of MHC antigen dependency to elicit adaptive antitumor immunity.

Song and Viskovska discussed the engineering of deimmunized vaccinia viral vectors using two strategies to weaken the strong anti-viral immune response that would eliminate the virus immaturely [21]. The first or main strategy is to mask the virus from the neutralization antibody responses by mapping and eliminating B-cell epitopes on the membrane proteins encoded by the viral genome. In a previous study, others have shown that the complement inhibitory protein DAF (CD55) suppresses T-cell immunity in vivo [22]. Therefore, in the second strategy, expressing the complement activation regulator CD55 on the surface of the virus particle would increase resistance to complement-mediated neutralization.

Everts, McFadden, and colleagues provided a comprehensive overview of the interactions between OVs and the different targetable tumor stromal components and outlined strategies to improve stroma targeting by OVs [23]. Tumor stroma is a multi-componential tissue consisting of mainly cancer-associated fibroblasts, the extracellular matrix, tumor vasculature, and tumor-associated macrophages. These components can all exert crucial roles in maintaining a pro-tumoral niche and are all potential targets for cancer therapy in which OVs can be a useful tool.

Finally, the team of Chaurasiya, Fong, and Warner reviewed the clinical experience of oncolytic virotherapy [24]. They focused on recent clinical studies with oncolytic HSV, VV, adenovirus, and reovirus. To summarize the current status of the clinical experience in one sentence, the OVs tested so far are well tolerated, but the therapeutic efficacies are modest. Therefore, the potency needs to be enhanced before more OVs hit the clinic. Alternatively, optimized combination strategies are highly desired.

## 3. Challenges of Oncolytic Immunotherapy

Since the previous Special Issue on Oncolytic Viruses in this journal four years ago [11], striking progress has been made. For example, one big limitation is that OVs need to be delivered intratumorally in order to achieve significant efficacy in cancer patients. Towards a feasible solution to deliver an OV systemically, Wang and his team have come up with a solution. They showed that transient inhibition of PI3Kδ enhances the therapeutic efficacy of an oncolytic VV delivered intravenously [25]. However, a number of the major hurdles still exist. Low efficiencies of both viral delivery to the tumor tissues and viral replication throughout the entire tumor tissue remains a challenge. These properties severely restrict the magnitude of therapeutic efficacy. Another hurdle is that the tumor microenvironment is highly immunosuppressive, and most OVs may not be able to modulate this effectively into pro-antitumor immunity. The studies published in this Special Issue II may provide some solutions to these challenges. In addition, a combination regimen involving OVs may provide solutions to this issue by turning cold tumors hot [7,8,9,10]. With active ongoing research and endeavors from many investigators, the future of these cutting-edge biomedicines looks much brighter.

## Figures and Tables

**Figure 1:**
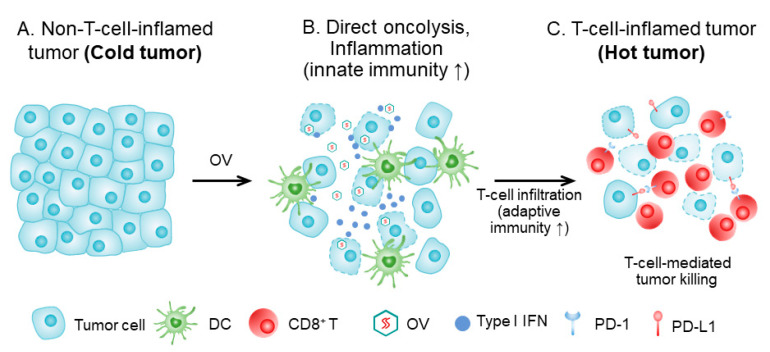
How OV turns a cold tumor hot. (**A**) Tumor tissue prior to OV treatment, with cancer cells (and stromal cells), lacking immune infiltrate (‘cold’ tumor). (**B**) The tumor tissue shortly after OV treatment. Infected tumor cells are killed by OV and the virus spreads to other tumor cells. Direct oncolysis and antigen presentation, and inflammation, lead to attraction of innate immune cells, and dendritic cells (DC) are attracted, matured, and activated. DCs take up dying tumor cells and present tumor antigens in the draining lymph node to tumor-specific T cells. (**C**). T cells are activated, proliferate, and home to the tumor tissue. The T-cell-inflamed tumor is a ‘hot’ tumor. This figure is adapted with slight modification from Oh, CM et al., 2020 [8], and used with permission from the authors.
